# Assessment of microsatellite instability status for the prediction of metachronous recurrence after initial endoscopic submucosal dissection for early gastric cancer

**DOI:** 10.1038/sj.bjc.6603532

**Published:** 2006-12-19

**Authors:** T Hasuo, S Semba, D Li, Y Omori, D Shirasaka, N Aoyama, H Yokozaki

**Affiliations:** 1 Division of Surgical Pathology, Department of Biomedical Informatics, Kobe University Graduate School of Medicine, Kobe, Japan; 2 Department of Endoscopy and Division of Diabetes, Digestive and Kidney Diseases, Kobe University Graduate School of Medicine, Kobe, Japan; 3 Division of Cellular and Molecular Medicine, Kobe University Graduate School of Medicine, Kobe, Japan

**Keywords:** microsatellite instability (MSI), early gastric cancer (EGC), endoscopic submucosal dissection (ESD), metachronous recurrent cancer

## Abstract

The technique of endoscopic submucosal dissection (ESD) has been developed for *en bloc* resection of early gastric cancer (EGC); however, little is known about the risk of metachronous cancer in the remnant stomach after initial ESD. In this study, we investigated the correlation between microsatellite instability (MSI) status and the incidence of metachronous recurrence of gastric cancer. According to the genetic/molecular background determined with MSI status and expression levels of hMLH1 and p53 tumour suppressor, 110 EGCs removed with ESD were subclassified into three groups: the mutator/MSI-type (8%), suppressor/p53-type (45%) and unclassified type (47%). Interestingly, patients with the mutator/MSI-type tumour had a high incidence (67%) of metachronous recurrence of gastric cancer within a 3-year observation after initial ESD, which was significantly higher than those with the suppressor/p53-type and unclassified type tumours (*P*<0.01). Although we investigated mucin phenotypes, there was no correlation between mucin phenotype and the recurrence of EGC. These findings suggest that subclassification of molecular pathological pathways in EGCs is required for the assessment of patients with a high risk of recurrent gastric cancer. The information delivered from our investigation is expected to be of value for decisions about therapy and surveillance after ESD.

Gastric cancer is the second-most-frequent malignant tumour worldwide, but the consequences of early gastric cancer (EGC) are comparatively good (Correa, 1992). In particular, endoscopic treatment is being widely used to treat patients with EGC and carries with it some improvement for their prognosis; therefore, many patients suffering from EGC have avoided laparotomy and maintained a better quality of life (Nagano *et al*, 2005). Endoscopic resection of EGC is now the standard therapy in Japan and is increasingly becoming accepted and regularly used in other countries. Recently, an innovational technique for endoscopic submucosal dissection (ESD) has been developed for *en bloc* resection of ECG (Ono *et al*, 2001). In addition, the ESD method has made it possible to give an accurate diagnosis by pathological examination (Toyonaga *et al*, 2006). However, metachronous recurrence at other sites in the stomach, after initial endoscopic treatment, has been identified as a major problem (Hamanaka and Gotoda, 2005).

Based on the tendency of gland formation, gastric carcinomas have been divided into two main histological types: diffuse-type carcinomas, which may arise from native gastric epithelial cells, and intestinal-type carcinomas, which were thought to arise from gastric epithelial cells that have undergone intestinal metaplasia (Lauren, 1965; Nakamura *et al*, 1968; Tahara, 1995). Current accepted indications for ESD of EGC include the resection of small intramucosal EGC of the intestinal type; the development of ESD techniques enables resection of larger lesions than has been technically feasible by simple endoscopic mucosal resection (EMR). However, the rationale behind this recommendation was that larger size lesions, or lesions with diffuse histology type, may extend into the submucosal layer and thus have a higher risk of lymph node metastasis. Therefore, at present, the accepted indications for ESD are: (i) well-differentiated elevated cancers less than 2 cm in size and (ii) small (⩽1 cm) depressed lesions without ulceration (Japanese Gastric Cancer Association, 2001). These lesions must also be moderately or well differentiated and confined to the mucosa, and must not have lymphatic or vascular involvement; well-differentiated type adenocarcinomas correspond to the intestinal-type (Lauren, 1965), the expanding-type (Ming, 1977) and the differentiated carcinoma (Nakamura *et al*, 1968).

Recent molecular biological studies have revealed that there are at least two distinct genetic pathways for gastrointestinal carcinogenesis, the suppressor and mutator pathways (Parsons *et al*, 1993; Eshleman and Markowitz, 1996). The suppressor/p53 pathway is mainly caused by inactivation of tumour suppressor genes and the activation of oncogenes. *p53* is a tumour suppressor that is frequently mutated in human cancer and is believed to play an important role in defending against cancer (Tamura *et al*, 1995). Microsatellite instability (MSI) is closely correlated with genetic instability, particularly in hereditary nonpolyposis colorectal cancer (HNPCC) (Fishel *et al*, 1993; Peltomalo *et al*, 1993). Dysfunction of the DNA mismatch repair system leads to the mutator pathway showing MSI; thus, MSI can be identified in tumours when alleles of novel sizes are detected in microsatellite sequences derived from cancer DNA that are not present in the normal tissue of the same individual. It has been documented that 15–33% of human gastric cancers exhibit MSI and that MSI is frequently observed in well-differentiated adenocarcinoma (Mironov *et al*, 1994; Semba *et al*, 1996). Furthermore, MSI was observed more frequently in patients with synchronous multiple gastric tumours than in those with a single tumour (Nakashima *et al*, 1995).

In this study, we focused on the metachronous recurrence of gastric cancer after removal of EGC (well-differentiated type) with ESD. After initial dissection, we subclassified these patients into three groups according to molecular findings and followed them endoscopically for several years to monitor the recurrence of gastric cancer.

## MATERIALS AND METHODS

### Patients and DNA extraction

The series consisted of 110 patients who had undergone curative resections for EGC with ESD at the Kobe University Hospital from 2000 to 2004. Their clinical and histological characteristics are summarised in Table 1. Informed consent for the scientific analysis of resected specimens was obtained from all patients, including those who had no hereditary cancer syndrome history, such as HNPCC, Li-Fraumeni syndrome and familial adenomatous polyposis (FAP), and those who had hereditary diffuse gastric cancer (HDGC). All resected specimens were fixed in 10% formalin and embedded in paraffin. Histopathological examination was performed according to the Japanese Classification of Gastric Carcinoma 2nd edition (Japanese Gastric Cancer Association, 2001). Early gastric cancers were defined as cancers in which invasion was limited to the submucosal layer, irrespective of the presence or absence of metastasis to lymph nodes. To exclude the incidence of local recurrence after initial ESD treatment, the histological curative resection was determined as follows; depth M (mucosa), histologically well differentiated adenocarcinoma (tub1 or tub2), neither lymphatic nor venous invasion, no tumour cells within 1 mm of lateral margin (1 mm lateral margin approximately corresponds to the length of ten tubules). The cases positive for lateral or vertical margins were therefore avoided in this study.

Selected blocks were sectioned into 4 *μ*m-thick slices for DNA extraction and immunohistochemical analysis. For DNA extraction, the deparaffinised specimens were stained with haematoxylin and eosin (H&E). The tumour areas and corresponding non-neoplastic gastric mucosa were scraped using a sterile needle and placed in a microtube containing extraction buffer before being incubated at 55°C for 12 h as described elsewhere (Wu *et al*, 2004). Proteinase K was inactivated by boiling for 5 min after inactivation and then the DNAs were used for polymerase chain reaction (PCR).

### Immunohistochemistry

Immunohistochemical analyses were carried out with monoclonal antibodies to p53 (DakoCytomation, Copenhagen, Denmark), hMLH1 (BD Biosciences, San Diego, CA, USA), CD10 (Novocastra Laboratories, Newcastle-upon-Tyne, UK), MUC2 (Santa Cruz Biotechnology, Santa Cruz, CA, USA), MUC5AC (Novocastra Laboratories) and MUC6 (Novocastra Laboratories). Dewaxed and rehydrated specimens were autoclaved in citrate buffer for the antigen retrieval. Endogenous peroxidase activity and nonspecific binding were blocked by 0.03% hydrogen peroxidase in methanol and blocking reagent in LSAB2 kit (DakoCytomation), respectively. The slides were then incubated with the primary monoclonal antibody and incubated sequentially with a biotinylated secondary antibody, streptavidin, labelled with peroxidase and 3, 3′-diaminobenzidine. Slides were counterstained with Mayer's haematoxylin. Immunoreactivity of p53 and hMLH1 was graded as follows: almost no positive cells; +, <25% of the tumour cells showed positive immunoreactivity; ++, 25–50% of the tumour cells showed positive immunoreactivity; +++, >50% of the tumour cells showed positive immunoreactivity (Li *et al*, 2005).

### Microsatellite analysis

Nine microsatellite markers (BAT-25, BAT-26, BAT-40, BAT-RII, D1S191, D2S123, D5S346, D17S250 and D17S855) were analysed (Li *et al*, 2005). The forward primers were fluorescein labelled with 6-FAM (D1S191, D17S250, BAT-26 and BAT-40), HEX (D17S855 and BAT-RII), VIC (D5S346) and TAMRA (D2S123 and BAT-25). PCR was performed in 15 *μ*l reaction volumes containing 1 *μ*l template DNA, 0.56 *μ*mol l^−1^ of each primer, 74.7 *μ*mol l^−1^ of dNTP, 4.5 *μ*mol l^−1^ of MgCl_2_ and 0.075 U of Ampli*Taq* Gold (Applied Biosystems, Foster City, CA, USA). After the initial *Taq* DNA polymerase activation step, the PCR amplification consisted of 45 cycles (94°C for 45 s, 55°C for 45 s and 72°C for 45 s) followed by a final extension for 10 min at 72°C. Polymerase chain reaction products were electrophoresed in ABI PRISM 310 Genetic Analyzer along with GeneScan-500 (ROX) molecular weight standard (Applied Biosystems). The size of the PCR product was analysed using GeneScan software (Applied Biosystems). The status of MSI in each tumour was evaluated according to the criterion of Boland *et al* (1998); MSI-H, if three or more out of nine microsatellite loci showed MSI; MSI-L, if one or two loci had MSI; MSS, if all the microsatellite loci examined were stable. Alleles were defined as the two highest peaks (tumour DNA alleles, T1, T2; normal DNA alleles, N1, N2) and a ratio of (T1/T2)/(N1/N2) of <0.67 or >1.50 was scored as a loss of heterozygosity (LOH) (Wu *et al*, 2004).

### Subclassification of mucin phenotypes

The extent of positivity for MUC2, MUC5AC, MUC6 and CD10 was scored according to the percentage of neoplastic cells stained: −, no positive cells or essentially none (<5%); +, some positive cells (5–30%); ++, well-defined areas of positive cells (30–60%); +++, extensive areas of positive cells (>60%). According to the sums of scores of gastric markers (MUC5AC, MUC6) and intestinal markers (MUC2, CD10), each case was phenotypically classified into three phenotypes: the gastric phenotype, mixed phenotype and intestinal phenotype (Tsukashita *et al*, 2001).

### Statistical analysis

Statistical comparisons were performed using the Fisher's protected least significant difference test, the *χ*^2^ test, the log-rank test and the Kaplan–Meier test for three independent groups. *P*-values of 0.05 or less were considered to indicate statistical significance. All statistical analyses were performed using Statview 5.0 software (SAS Institute Inc., Cary, NC, USA).

## RESULTS

### Subclassification of EGC with genetic background: the mutator/MSI-type and suppressor/p53-type

**Figure 1:**
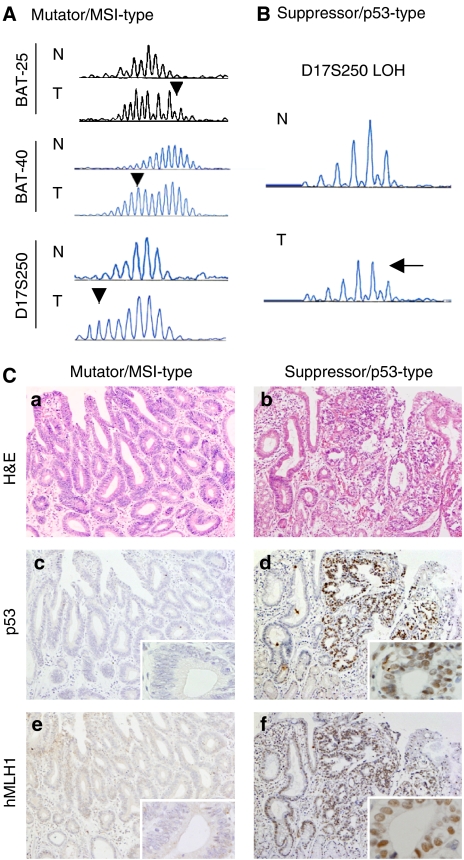
Representative results of microsatellite assay and immunohistochemistry. (**A**) Results of microsatellite analysis on BAT-25, BAT-40 and D17S250. Arrowheads indicate MSI. N, normal mucosa; T, tumour. (**B**) Loss of heterozygosity at the *p53* locus (D17S250). Decreased peak levels in tumour are regarded as LOH (arrow). N, normal mucosa; T, tumour. (**C**) Histological examination of EGCs. Representative mutator/MSI-type (a, c, e; × 100) and suppressor/p53-type (b, d, f; × 100) tumours are illustrated. a–b, H&E.; c–d, p53; d–f, hMLH1 (inset original magnification × 400).

**Figure 2:**
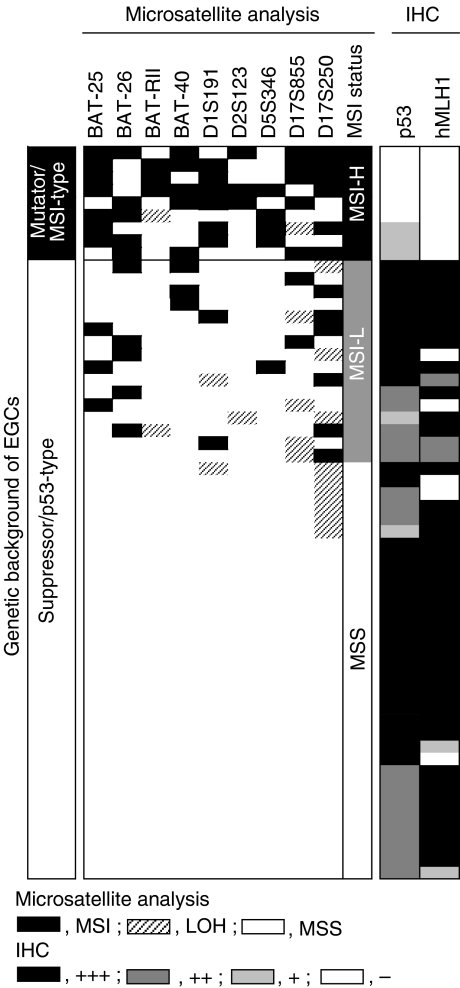
Results of microsatellite assay and immunohistochemical analyses (IHC) in the mutator/MSI-type and suppressor/p53-type human EGCs. The status of microsatellite sequences was determined as described in the text. Immunoreactivity of p53 and hMLH1 was graded from − to +++. According to these results, tumours were subclassified into the mutator/MSI-type, suppressor/p53-type, and unclassified type tumours.

To determine the genetic background of each tumour, we first performed a microsatellite assay in tumours with microsatellite markers (Figure 1A). Overall, MSI was detected in 25 (23%) of 110 cases (MSI-H, nine (8%) cases; MSI-L, 16 (15%) cases) and LOH at the *p53* locus (D17S250) was detected in eight (7%) of 110 cases (Figure 1B and 2). Next, we investigated the levels of p53 and hMLH1 expression immunohistochemically to distinguish the mutator/MSI-type tumours and suppressor/p53-type tumours. Strong nuclear accumulation of p53 (++, +++) was detected in 47 (43%) cases and loss of hMLH1 expression (−) was detected in 28 (25%) cases, respectively (Figures 1C and 2).

Two distinct pathways, the mutator and suppressor pathways, have been proposed for colorectal carcinogenesis (Parsons *et al*, 1993). Similarly, this concept is believed to be available for subclassification of human gastric cancers (Yamamoto *et al*, 1995; Semba *et al*, 1996; Endoh *et al*, 2000; Furlan *et al*, 2002; Wu *et al*, 2004). Therefore, we attempted to subclassify these EGCs with the definition of the mutator (mutator/MSI-type) and suppressor (suppressor/p53-type) pathways (Eshleman and Markowitz, 1996). According to the results of genetic and immunohistochemical analyses, we therefore subclassified these EGCs into three subtypes: the mutator/MSI-type (tumours with MSI-H and loss of hMLH1 expression), nine (8%) cases; suppressor/p53-type (tumour with strong p53 expression, or with LOH at D17S250), 49 (45%) cases and unclassified type, 52 (47%) cases (Figure 2 and Table 2). All of the cases of the mutator/MSI-type tumours showed loss of hMLH1 expression, whereas 41 of 49 (84%) and 27 of 52 (52%) cases in the suppressor/p53 and unclassified types had strong expression of hMLH1. No significant correlation was found between genetic type and clinicopathological parameters.

### Expression of epithelial mucin in EGCs

We investigated mucin expression in these EGCs because a previous study reported that subclassification of gastric cancer mucin is likely to be useful in predicting the pattern of gastric carcinoma recurrence after curative surgery (Tajima *et al*, 2004). According to the results of immunohistochemical analyses of epithelial mucin expression, the EGCs were classified into three mucin phenotypes: the gastric phenotype, 50 (46%) cases; mixed phenotype, 17 (15%) cases and intestinal phenotype, 43 (39%) cases. There was significant difference between mucin phenotypes and location (*P*=0.007), but no correlation was found in the other clinicopathological parameters (Table 2).

### MSI status is a good marker for the possible development of metachronous cancer

**Figure 3:**
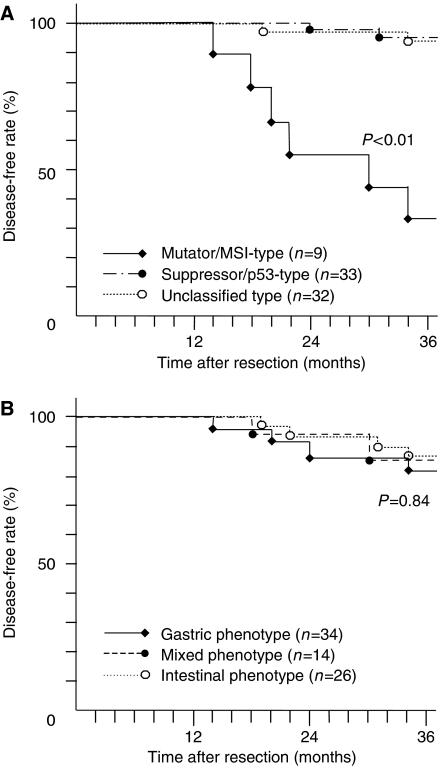
Kaplan–Meier curves for ECG patients who had metachronous cancer after initial ESD treatment. (**A**) Overall disease-free curves in relation to the mutator/suppressor subtypes in patients with EGCs. The 3-year disease-free rate in the group of mutator/MSI-type tumours was 33%, but it was 93 and 94% in the suppressor/p53-type and unclassified type tumours, respectively (*P*<0.01). (**B**) Overall disease-free curves in relation to mucin subtypes in patients with EGCs. There was no statistical difference in the 3-year disease-free rate among mucin phenotypes (*P*=0.84).

The patients examined were regularly followed and observed endoscopically to monitor for secondary metachronous tumours in the remnant stomach after ESD. Interestingly, patients with mutator/MSI-type EGCs had a tendency to develop secondary metachronous cancer in the remnant stomach after initial ESD, which was statistically higher than the suppressor/p53 and unclassified type tumours (*P*<0.01, Figure 3A), whereas no correlation was found in the increase of metachronous gastric cancers by subclassification of tumours with mucin phenotypes (*P*=0.84, Figure 3B). We also investigated MSI and p53 and hMLH1 expression in the metachronous secondary gastric cancers; all secondary tumours arising from patients with the mutator/MSI-type EGC also showed MSI and loss of hMLH1 expression (data not shown).

## DISCUSSION

Endoscopic treatment for patients with early-stage gastric carcinoma has been widely used for curative therapy, and such endoscopic tumour resection has enabled patients with gastric cancer to have better prognoses (Haruma *et al*, 1990; Sano *et al*, 1992; Amano *et al*, 1998). Although ESD is a useful method newly developed not only for endoscopic curative resection of EGCs but also for accurate histological diagnosis, the indications for such endoscopic treatment are limited to small, well-differentiated adenocarcinomas with minimal submucosal invasion (Hirasaki *et al*, 2005; Kato, 2005). Endoscopic treatment excised only gastric carcinoma tissues, which implies that patients are still at risk of developing metachronous recurrence of gastric cancer. Therefore, molecular diagnosis is required for the screening of patients expected to have metachronous gastric carcinoma (Miyoshi *et al*, 2001).

This is the first report indicating the clinical usefulness of microsatellite analysis for predicting the potential risk of developing metachronous gastric cancer after curative resection with ESD. Miyoshi *et al* (2001) examined MSI retrospectively in patients with multiple EGCs and found that MSI increased the frequency of synchronous and metachronous gastric cancer development. However, gastric carcinoma tissues removed with EMR may possibly be related to local recurrence of carcinoma, and researchers collected multiple EGC tissues with the intention of analysing the relationship between MSI and synchronous and metachronous gastric cancer. In the current study, we could eliminate the local recurrence of gastric cancer because ESD is a more efficient method for curative resection of EGC and enables more accurate histopathological diagnosis than EMR. In addition, our cohort study indicated a high frequency of MSI linked with recurrence of carcinoma in patients with EGC. Thus, subclassification of EGCs into two distinct groups, the mutator/MSI-type and the suppressor/p53-type, may help clinically in defining a molecular marker for the prediction of multiple and metachronous gastric cancer.

Correa (1992) hypothesised that carcinogenesis of the stomach is a multistage process, and the progression from normal epithelial cells to tumour cells involves at least six stages: superficial gastritis, chronic atrophic gastritis, intestinal metaplasia of the complete type followed by the incomplete type, gastric adenoma, dysplasia and carcinoma. Although chronic infection of *Helicobacter pylori* (*H. pylori*) (Blaser, 1987) and excess salt and nitrosamine compound intake (Sugimura and Wakabayashi, 1990) are suspected to be important causal factors in gastric carcinogens, little is known about the mechanism through which the incidence of gastric cancer might be increased. In this study, infection of *H. pylori* was confirmed in all cases, and eradication treatment was performed before or after ESD; the eradication therapeutic success rate was 95%, and a second eradication treatment using another antibiotic was performed in the failure group (data not shown). The data suggest that chronic atrophic gastritis with or without intestinal metaplasia, due to *H. pylori* infection, certainly increases the incidence of gastric cancer, irrespective of the mutator/MSI-type or the suppressor/p53-type. However, further genetic changes may finally decide the carcinogenesis pathway for epithelial cells to develop gastric cancer. As reported previously, an MSI-positive genetic background is a risk for developing multiple cancers, including synchronous and metachronous cancer (Miyoshi *et al*, 2001). As our study indicated that patients with the mutator/MSI-type EGC are expected to have a higher risk of developing metachronous mutator/MSI-type tumours in the stomach, the assessment of MSI is necessary for following up these patients with MSI-positive EGC.

Tajima *et al* (2004) showed that the incidence of gastric mucin-producing tumours was significantly higher in tumours with recurrence than in tumours without recurrence after curative surgery, irrespective of their histological type. Their study was performed using advanced cancer; presumably, the results may have been influenced by the other characteristic factors of cancer cells, including resistance to chemotherapy, growth rate and vessel infiltration activity. No significant correlation was found between MSI and mucin phenotype in this series of EGC, in which most tumours demonstrated well-differentiated tubular formation (tub1 and tub2), suggesting that recurrence of gastric cancer may not be controlled by mucin phenotype (Shibata *et al*, 2003). Although it has also been documented that mucin phenotype may be associated with MSI status (Endoh *et al*, 2000; Takahashi *et al*, 2002), our results indicate the opposite conclusion; mucin phenotypes did not connect with genetic alterations in microsatellite loci. Further investigation is required to clarify this discrepancy.

## Figures and Tables

**Table 1:** Clinical and histological characteristics of early gastric cancers

**Characteristic**	**Group**	**No.**	**(%)**
Gender	Male	74	(67)
	Female	36	(33)
Age^a^(year)	<65	53	(48)
	⩾65	57	(52)
Tumour size (cm)	<2	67	(61)
	⩾2	43	(39)
Location^b^	Upper	30	(27)
	Middle	31	(28)
	Lower	49	(45)
Differentiation^b^	tub1	84	(76)
	tub2	26	(24)

a Average age (years); 65.3±7.3.

b Location of tumours and histological differentiation were determined according to the Japanese Classification of Gastric Cancer 2nd edition. tub1=well-differentiated tubular adenocarcinoma, tub2=moderately differentiated tubular adenocarcinoma.

**Table 2:** Relationship between genetic type/mucin phenotype and clinicopathological parameters in early gastric cancers

	**Genetic type**				**Mucin phenotype**			
	**Mutator/MSI**	**Suppressor/p53**	**Unclassified**	***P*-value**	**Gastric**	**Mixed**	**Intestinal**	***P*-value**
*Gender*								
Male	6	32	36		34	11	29	
Female	3	17	16	0.931	16	6	14	0.989
								
*Age (years)*								
<65	4	20	29		23	7	23	
⩾65	5	29	23	0.424	27	10	20	0.804
								
*Tumour size (cm)*								
<2	7	30	30		30	9	28	
⩾2	2	19	21	0.774	20	8	15	0.840
								
*Location* a								
Upper	2	12	14		20	4	6	
Middle	4	13	14		16	7	8	
Lower	3	24	24	0.969	14	6	29	0.007^*^
								
*Differentiation* a								
tub1	7	38	39		34	13	37	
tub2	2	11	13	0.956	16	4	6	0.192
								
Total (%)	9 (8)	49 (45)	52 (47)		50 (46)	17 (15)	43 (39)	

a Location and histological differentiation of the tumours were determined according to the Japanese Classification of Gastric Cancer 2nd edition (2001). tub1= well-differentiated tubular adenocarcinoma, tub2= moderately differentiated tubular adenocarcinoma.

^*^There was significant difference between mucin phenotypes and location of EGC (*P*=0.007, Fisher's test).
